# Effect of Bias Arc on Microstructure and Corrosion Resistance of Q235/304 Dissimilar-Steel-Welded Joints

**DOI:** 10.3390/ma17174234

**Published:** 2024-08-27

**Authors:** Lin Li, Rongcai Ma, Cheng Yang, Tie Liu, Guorui Sun, Wenlong Li, Chuanchuan Jia, Chao Chen, Fengya Hu

**Affiliations:** 1 State Key Laboratory of Metal Material for Marine Equipment and Application, Anshan 114003, China; lilin2021@126.com; 2 Research Institute of Ansteel Group, Anshan 114003, China; yangcheng@163.com; 3 Ansteel Group Multi-Industry Development Co., Ltd., Anshan 114011, China; rongcaimaanshan@126.com; 4 Steel Making General Plant of Angang Steel Co., Anshan 114021, China; tieliu2018@126.com; 5 Key Laboratory of Automobile Materials, School of Materials Science and Engineering, Jilin University, Changchun 130025, China; guoruisun2020@126.com (G.S.); liwenlong2022@126.com (W.L.); jiachuanchuan2019@126.com (C.J.)

**Keywords:** dissimilar welding, bias arc, microstructure, electrochemistry, corrosion resistance

## Abstract

To fully exploit the advantages of steel, the welding connection of dissimilar steels has been developed. In this work, the metallographic microstructures, elemental distributions, and electrochemical corrosion properties of the Q235 and 304 welds under different bias arcs were investigated. The arc bias caused the Q235-side heat-affected zone to widen, the microstructure consisted of ferrite and pearlite, and the ratio varied with decreasing distance from the fusion line. Elemental scans show that Cr and Ni concentration gradients exist near the fusion line. The 304-stainless-steel-side heat-affected zone was mainly composed of austenite grains, and the fusion zone was narrower but prone to cracking. Electrochemical tests revealed that 304 stainless steel had the best corrosion resistance, while Q235 had the worst corrosion resistance, and that the welded joints with an arc bias toward the 304 side had the best corrosion resistance. The samples’ the passivation film which formed via electrochemical polarization had limited stability, but the over-passivation potential could be used as a reference for corrosion resistance. Overall, the arc bias and weld material properties significantly affected the microstructure and corrosion resistance of the joints.

## 1. Introduction

The most commonly used stainless steel material is 304 stainless steel; it has excellent plasticity, toughness, corrosion resistance, and other comprehensive mechanical properties and is widely used in the shipping, aviation, chemical, and pharmaceutical fields [1,2,3]. Its thermal conductivity is small, and the coefficient of linear expansion is large [4]. Q235 steel is a commonly used low-carbon steel with a relatively low price and excellent welding performance and is a common steel in actual production. Owing to its low carbon content, the manganese and silicon content of Q235 steel is also low, and it is usually welded without the hardening or embrittlement of its microstructure; additionally, the impact toughness and plasticity of the welded joints are better, and the interlayer temperature is not preheated or controlled. No heat treatment is required after welding, and the welding performance is excellent [5,6,7]. However, compared with stainless steel, Q235 steel has poor corrosion performance, which largely limits its application [8,9]. WANG et al. [10] prepared a 304-stainless-steel-cladding layer on the surface of Q235, and the corrosion resistance of the cladding layer was significantly greater than that of the Q235 substrate.

With the rapid development of modern industry and the use of metal materials in increasingly complex environments, traditional single metals have difficulty meeting harsh external conditions [11]. Welded structural components of dissimilar steels combine the performance advantages of different steels and reduce production costs on the basis of guaranteed performance, which has been widely used in petrochemicals, aerospace, shipbuilding, power station boilers, and many other industrial fields [12]. Dissimilar steel welding not only meets the requirements of welded joints in complex working conditions but also reduces the production cost of the equipment. XU et al. [6] investigated the microstructural evolution and mechanical properties of Q235 steel and 304 stainless steel joints. The results revealed that the microstructure of the weld zone (WZ) of the Q235 steel consisted of slat martensite with carbon migration at the interface. The tensile samples fractured on the Q235 steel side, indicating that the welded joints were stronger than those of the Q235 base material (BM). These fracture patterns were characterized by ductile fracture. The distribution of the microhardness in various regions of the welded joints was as follows: WZ > 304 side HAZ > 304 BM > Q235 side HAZ > Q235 BM. WANG et al. [13] investigated the effect of the microstructure on the corrosion behavior of dissimilar friction-stir-welded (FSW) joints of 304 stainless steel (SS 304) and Q235 mild steel. A large number of low-angle grain boundaries (LAGBs) and a low percentage of double grain boundaries (TBs) reduced the corrosion resistance of the SS 304 side of the joint.

Tungsten inert gas shielded welding (TIG) is the most commonly used welding method. In the arc-welding process, the thermal properties and permeability of the Q235 mild steel and 304 stainless steel arc are different from those of the mild steel side, resulting in an unstable welding process, poor weld formation, and a reduced depth of fusion [14]. Differences in the thermal properties of dissimilar steels, such as their thermal conductivity, specific heat capacity, and melting point, indicate that the heat input to the base material on both sides should differ. Many problems can arise during the welding of dissimilar steels [15,16]. In this case, conventional symmetrical heat input may lead to welding defects such as poor depth of fusion, stress concentration, and even cracks. ZHAO et al. [17] investigated the GTAW arc energy distribution law for Q235 and 304 L dissimilar steel filet welds. When the arc is in the center of the weld, the GTAW arc deflects toward the Q235 steel side because the relative permeability of the Q235 steel at room temperature is 285, whereas the relative permeability of the 304 stainless steel is 1 [18]. Owing to the better magnetic conductivity of the Q235 material, the arc magnetic flux density on the Q235 side is reduced so that the arc magnetic field strength on the Q235 side is less than that on the 304 stainless steel side, and the arc force is less than that on the 304 stainless steel side; so, the arc deflects to the Q235 side, which increases the melting width on the Q235 side. WANG et al. [19] investigated the effect of laser beam offset on the microstructure and properties of laser-welded joints of Q235/304 stainless steel. The results showed that when the laser beam was biased toward the Q235 side, the molding quality was better, the martensite content in the heat-affected zone was greater, and the grains were fine and uniform. When the laser beam was biased toward the 304 stainless steel side, the martensite content in the heat-affected zone of the Q235 side decreased, the ferrite content increased, and the grains grew sharply. The maximum hardness of the joints appeared in the weld region, which was 3.5 times greater than that of the Q235 base material. There was little difference in the tensile properties of the joints with different offsets, all of which broke on the Q235 side, indicating that the joints exceeded the strength of the Q235 base material. Owing to the high Cr content in SS 304, a dense oxide film can be formed; so, the corrosion resistance is excellent. The carbon content and alloy content of the Q235 low-carbon steel for pearlite carbon structural steel are lower, and the plasticity and toughness are better. The overall performance is better, but the corrosion resistance is worse than that of 304. 

The corrosion resistance of the welded joints directly affects the service life and safety of welded structures, especially important in highly corrosive environments such as marine and chemical industries. Arc bias improves the corrosion resistance of welded joints and extends the service life of structures by optimizing the microstructure and chemical composition of the weld. Select the appropriate bias parameters under different working conditions to achieve the desired results. Above all, owing to the different material properties of dissimilar steels, the content and morphology of the microstructure in the weld region will change significantly, which in turn will affect the strength and corrosion resistance of the joint. At present, there are few studies on the microstructure and corrosion performance of welded joints because of the offset position of the heat source of dissimilar welding. Q235 and 304 stainless steel are widely used in industrial production and have broad application prospects if their excellent properties can be combined. In this work, Q235/304 stainless steel dissimilarity welding is performed via GTAW. The effects of the welding arc position (weld center, biased 304 stainless steel side, and biased Q235 side) on the microstructure and corrosion properties were investigated to provide a new direction for Q235/304-stainless-steel-dissimilar-welding applications.

## 2. Experimental Materials and Methods

The welded plates selected for this study are 120 mm × 80 mm × 3 mm of 304 stainless steel and Q235 mild steel. Stainless steel 304 is a typical austenitic stainless steel. The chemical compositions of 304 and Q235 are shown in Table 1. Before welding, an angle grinder was used to clean up the oxidized skin, grease, dust, water, and pollution within 50 mm on each side of the docking surface and its sides, and the samples were cleaned with alcohol.

**Figure 1:**
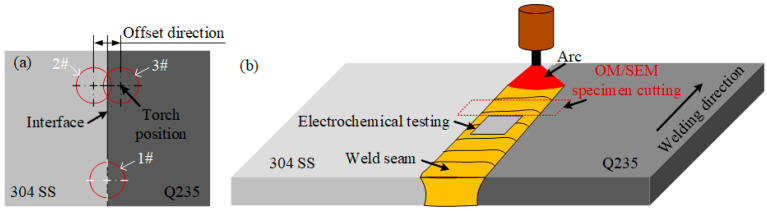
Q235/304 offset welding method: (**a**) top view; (**b**) schematic of welding process.

The welding method selected is unfilled Tungsten inert gas shielded welding (TIG). When heterogeneous materials are welded, the bias parameters of the arc have a greater impact on the microstructure and corrosion resistance of the weld. The amount of arc bias is set to 0, 0.5 mm, and −0.5 mm. No arc bias is marked as 1#, the arc bias 304 side is noted as 2#, and the arc bias Q235 side is noted as 3#. The 304/Q235 offset welding method is shown in Figure 1a. In the welding process, to control the welding process parameters as well as possible, the welding current was set to 180 A, the protective gas flow rate was 15 L/min, and the welding speed was 3 mm/s.

After the samples were cooled to room temperature after arc welding, metallographic (OM) samples and scanning electron microscopy (SEM) samples were prepared by using a CNC wire-cutting machine. The 7 mm × 7 mm electrochemical samples were cut at the center of the weld. After the metallographic specimens had been ground with 600–1200# sandpaper, they were then polished with a 0.5 μm diamond polish. After polishing, the metallographic specimens were corroded using aqua regia for 10 s and quickly rinsed with anhydrous ethanol. An Axio Scope A1 metallographic microscope (Oberkochen, Germany) and a TESCAN VEGA3 tungsten filament scanning electron microscope (Bron, Czech Republic) were used to observe the microstructure of the joint cross-sections, and an energy spectrometer was used to line-scan and surface-scan the welded joint elements, respectively.

The corrosion resistance of the weld surfaces was determined via a CHI660E electrochemical workstation (Shanghai, China) with an experimental solution of 3.5 wt.% NaCl prepared from deionized water and analytically pure chemical reagents. Open-circuit potential, kinetic potential polarization, and electrochemical impedance tests were performed in a three-electrode system. The working electrode was immersed in the test solution for 40 min before the start of the test, and the open-circuit potential was stabilized before the kinetic potential polarization test was performed. The electrochemical impedance spectra were scanned in the frequency range of 0.01 Hz to 100 kHz. The polarization curves were scanned at a rate of 1 mV/s with a scanning range of ±1.5 V. The polarization curves were obtained from the electrodes of the three-electrode system.

## 3. Results and Discussion

**Figure 2:**
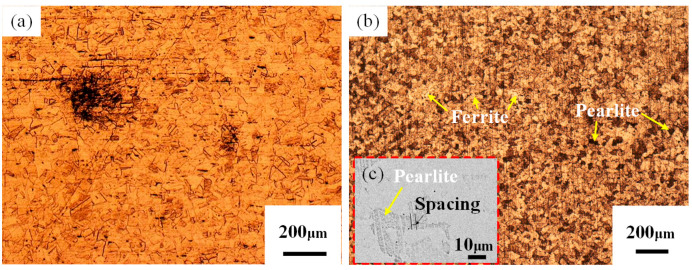
Microstructure of the base material: (**a**) BM_304_ OM map; (**b**) BM_Q235_ OM map; (**c**) BM_Q235_ SEM map.

Figure 2 clearly shows the characteristics of the base material microstructures of 304 stainless steel and Q235 steel. For 304 stainless steel (Figure 2a), the matrix microstructure is mainly composed of austenite; this austenite has relatively coarse grains, the grain microstructure is compact and uniform in size, and there are no precipitates at the grain boundaries. Notably, many twin structures exist within the austenitic grains, which are arranged along the rolling direction, and a small amount of residual high-temperature δ-ferrite is also visible, which helps increase the stability of the austenitic steel. The microstructure of the Q235 steel is mainly composed of ferrite and pearlite, as shown in Figure 2b. Ferrite appears as a gray polyhedral microstructure, whereas pearlite appears as a black polyhedron, which is relatively uniformly distributed in the ferrite grain boundaries and their interiors. Further observation of Figure 2c reveals that the pearlite has a typical lamellar morphology with lamellar spacings between 0.8 and 1.1 μm, in which the carburite is intermittently rod-like or granular.

### 3.1. Q235/Weld Interface Microstructure Analysis

**Figure 3:**
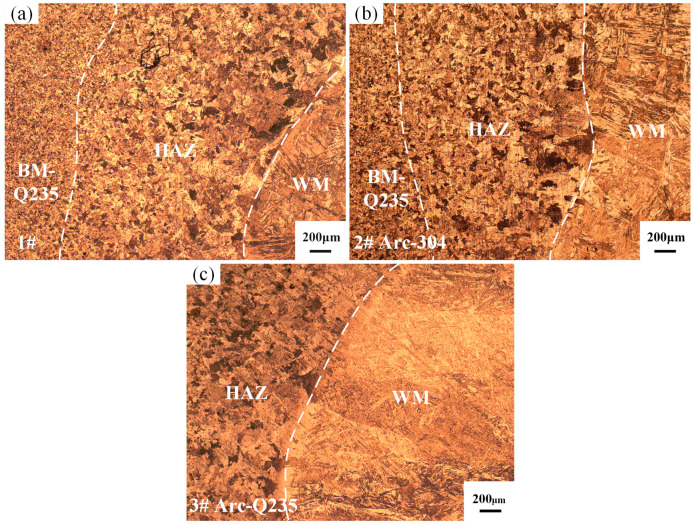
OM maps of the fusion line position on the Q235 side: (**a**) sample 1#; (**b**) sample 2#; (**c**) sample 3#.

Figure 3 shows the metallographic microstructure of the Q235/weld near the fusion line obtained under different bias arcs. Figure 3a shows that the location of the fusion line between the Q235 base metal and the weld metal is more pronounced and that there is a clear heat-affected zone. The heat-affected zone and the Q235 base metal are on the left side of the fusion line, and the weld seam is on the right side. Since the magnetic permeability of mild steel is greater than that of stainless steel, the arc will be biased more toward mild steel. During the welding process, even if the tungsten electrode is located directly above the weld seam, the arc will be more biased toward the Q235 side, the Q235 base metal will melt more than 304 stainless steel, and the heat-affected zone will be larger at the same time. The width of the heat-affected zone is approximately 2 mm for sample 1# and 1.7 mm for sample 2#. When the arc is biased to the Q235 side, the heat input to the Q235 base material is greater, the heat-affected zone is further expanded, as shown in Figure 3c, and the width of the heat-affected zone is greater than 2 mm.

The OM map reveals that the bias of the arc does not lead to changes in the microstructure of the heat-affected zone, which is mainly composed of ferrite (F) and pearlite (P), and this compositional ratio changes significantly with decreasing distance from the fusion line, as evidenced by a gradual decrease in the content of pearlite. Immediately to the left of the fusion line, there is a region of significant superheating, where carbides precipitate in an ordered manner within the austenite grains and tend to extend in a needle-like fashion; ultimately, these structures progressively develop into coarse Weiss tissue with a complex lattice or feathery texture. The evolution of the microstructure exhibits continuous characteristics, as evidenced by a clear downward trend in the amount of pearlite as the fusion line approaches. When the arc deflects to the 304 side, the heat-affected zone on the Q235 base metal side is small, and the proportion of pearlite in the heat-affected zone is larger and closer to the base metal than that of sample 1#. The edge of the molten pool is near the base metal because the liquid metal residence time is shorter, and the mechanical stirring effect is weakened; so, the composition of the weld in this region is closer to that of the base metal.

**Figure 4:**
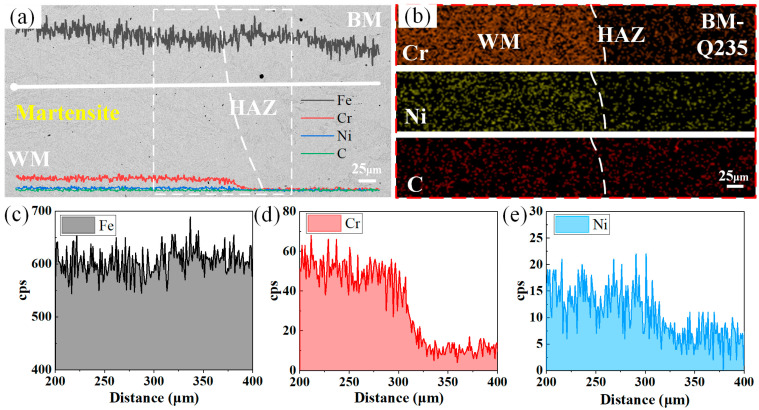
Elemental-scanning results: (**a**) SEM map of the scanning area; (**b**) elemental surface scanning; (**c**) elemental-line-scanning results for Fe; (**d**) elemental-line-scanning results for Cr; (**e**) elemental-line-scanning results for Ni.

Figure 4 shows the elemental-scanning results of the fusion line position on the Q235 side. The elemental-line-scanning analysis of the interface between the Q235 and the weld clearly reveals that key elements, such as Cr and Ni, exhibit significant concentration gradient changes in the region adjacent to the fusion line of the joint. Specifically, in the narrow region adjacent to the fusion line on the Q235 side of the joint, the fluctuation in the elemental concentration is relatively smooth, indicating a relatively uniform elemental distribution. However, once close to the fusion line on the weld side, the change in the elemental concentration becomes particularly drastic: the content of Cr and Ni increases dramatically, and this transition constitutes a significant concentration gradient interface.

In Figure 4a, we observe a significant increase in the content of Cr- and Ni-alloying elements as the scanning direction moves toward the center of the weld, especially for Cr, which significantly increases in content close to the fusion line. In contrast, the change in the content of Ni is slightly greater. This phenomenon is explained by the fact that the high Cr and Ni contents in the 304 stainless steel are diluted into the weld. Moreover, there was a slight decrease in the Fe content, which was caused by the increase in the Cr and Ni contents.

### 3.2. Weld/304Interface Microstructure Analysis

**Figure 5:**
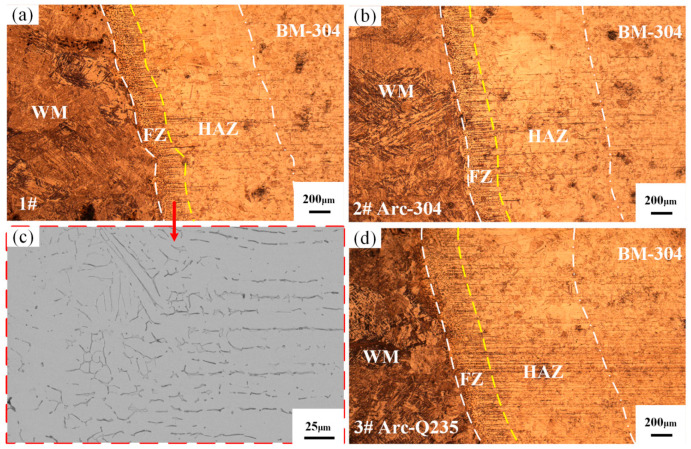
OM and SEM images of the fusion line location on the 304 side: (**a**) OM image of sample 1#; (**b**) OM image of sample 2#; (**c**) SEM image of the fusion zone; (**d**) OM image of sample 3#.

On the 304 stainless steel side shown in Figure 5, the location of the fusion line can be clearly observed via the OM and SEM map analysis. This region demonstrates that the heat-affected zone is mainly composed of austenitic grains, but the grain size distribution is not uniform. This nonuniformity stems from the differences in the degree of mixing of the weld metal with the base metal in the molten pool and at the edges when welding Q235 to 304 stainless steel. In particular, close to the edge of 304 stainless steel, the lower temperature, restricted metal mobility, and short liquid-residence time result in a unique fusion zone where the molten metal and base metal fail to fully fuse at the interface. As shown in the figure, the fusion zone is formed on the 304 side. The fusion zone is the weakest part of the welded joint and is prone to hot and cold cracks. In actual industrial production, because the main weak link of the joint is generally the fusion zone, it is necessary to strictly control the width of the fusion zone. During low-carbon steel welding, the welding-process-induced fusion zone is heated to the highest temperature, reaching 1400~1539 degrees. Celsius metal partially melts; after crystallization, the material does not melt but rather experiences heated growth of coarse grains and new crystallization of the cast. Figure 5 shows that the widths of the joints welded in joints No. 1 and No. 2 in the fusion zone are between 200 and 300 μm. The welded joints in the fusion zone of mild steel are very narrow, generally approximately 0.1~1 mm, but they largely determine the mechanical properties of the welded joints.

At the junction of 304 stainless steel and the weld, along the boundaries of the austenitic grain boundaries, there is a fine distribution and a discontinuous state of worm-like ferrite. Further observation of the heat-affected zone reveals that the microstructure consists mainly of austenitic equiaxial crystals, and banded ferrite is scattered on these grains. The size of these grains does not change significantly compared with that of the parent material, but the amount of banded ferrite in the heat-affected zone is significantly greater than that of the parent material.

When 304 stainless steel is welded with Q235 carbon steel, the enrichment of the weld Cr reduces the carbon activity, resulting in the tendency of carbon to diffuse from the Q235 to the weld. The SEM observations revealed the distribution of ferrite on the austenitic matrix near the fusion line. Ferrite can eliminate single-phase austenite directionality, refine grains, and reduce intergranular segregation, thus enhancing the crack resistance and intergranular corrosion resistance of welded joints, which is a key factor in improving the welding quality.

**Figure 6:**
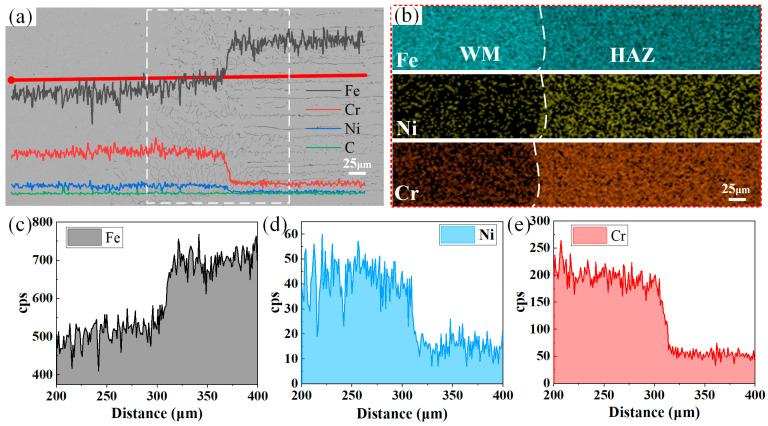
Elemental-scanning results: (**a**) SEM map of the scanning area; (**b**) elemental surface scanning; (**c**) elemental-line-scanning results for Fe; (**d**) elemental-line-scanning results for Ni; (**e**) elemental-line-scanning results for Cr.

Figure 6, on the other hand, shows the line scan direction opposite to the Q235 side, i.e., from the center of the weld toward the 304 stainless steel side. Along this path, the content of Cr- and Ni-alloying elements gradually increases, whereas the content of Fe decreases accordingly. This reflects the dilution of Cr- and Ni-alloying elements in 304 stainless steel by Q235 carbon steel during the dissimilar-steel-welding process.

### 3.3. Weld Metal Microstructure Analysis

**Figure 7:**
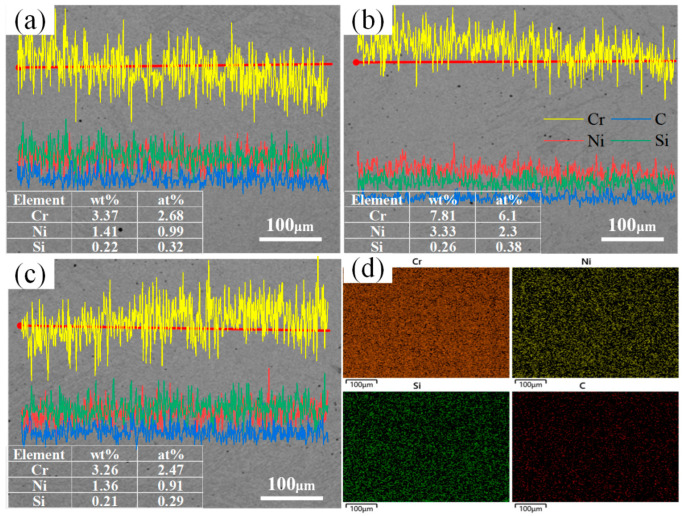
Weld-element-scanning results: (**a**) Line-scanning results for sample 1#; (**b**) line-scanning results for sample 2#; (**c**) line-scanning results for sample 3#; (**d**) surface scanning element distribution.

**Figure 8:**
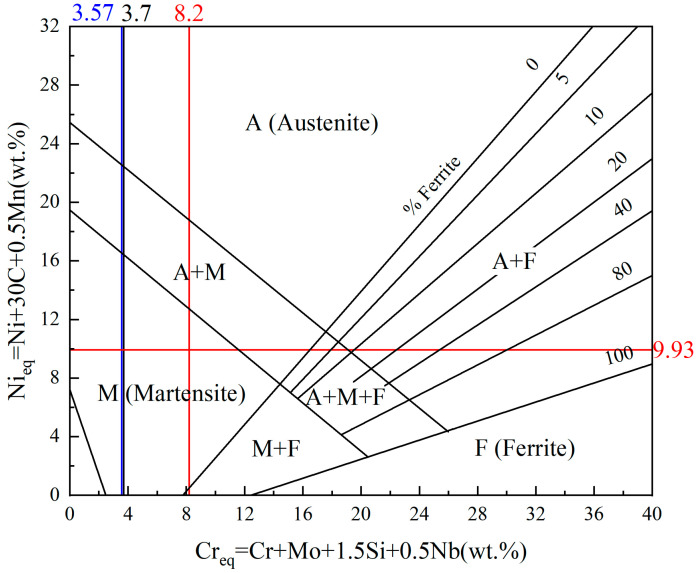
Schaeffler diagram.

According to the results of the elemental content test of the energy spectrometer, the Cr contents in the scanning regions of the 1#, 2#, and 3# welds were 3.37, 7.81, and 3.26, respectively, and the Ni contents were 1.41, 3.33, and 1.36, respectively, as shown in Figure 7. Combined with Formulas (1) and (2) of Cr-equivalent (Creq) and Ni-equivalent (Nieq) in Schaeffler’s phase diagram, the Cr equivalents in the weld regions of samples 1#, 2#, and 3# were 3.7, 8.2, and 3.57, respectively. The Nieq value was calculated to be a maximum of 9.93 on the basis of the maximum carbon content of 0.22 wt% in the Q235 plate. The results of the Creq and Nieq calculations can be combined with the Schaeffler diagram [20] (Figure 8) to determine that the microstructure within the welded joint is martensite.

Creq = Cr + Mo + 1.5Si + 0.5Nb(wt.%)
(1)

Nieq = Ni + 30C + 0.5Mn(wt.%)
(2)

**Figure 9:**
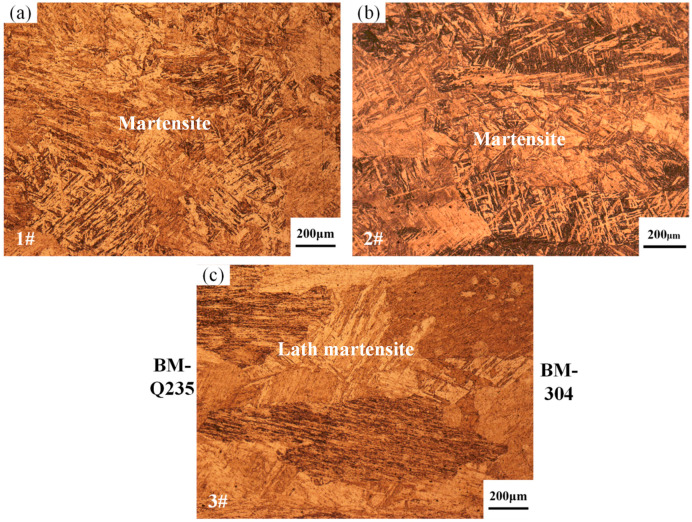
OM map of weld center position: (**a**) OM map of sample 1#; (**b**) OM map of sample 2#; (**c**) OM map of sample 3#.

The Q235 and 304 dissimilarity welding weld is a low-carbon steel; its weld-lath martensite formation originates from the specificity of the welding thermal cycle, and rapid cooling leads to nonequilibrium solidification. The rapid cooling process of steel in the austenitic state of diffusion-type decomposition is inhibited, a diffusion-free phase transition process occurs, and martensite microstructure is eventually formed at room temperature. The weld-center microstructure verified the theoretical predictions. However, stainless steel welding is prone to produce harmful precipitates, such as the σ-phase and M_23_C_6_ phases, at 427~800 °C, which originates from the sensitization phenomenon of chromium-nickel austenitic stainless steels, leading to chromium depletion at the grain boundaries and a reduction in corrosion resistance. Compared with the 1# sample, the 3# sample with the arc bias toward the Q235 side exhibited fine martensite bars of approximately the same size oriented in parallel, forming large martensite bundles, i.e., lath martensite. The 2# sample with the arc bias toward the 304 side formed a fine, needle-shaped, and lath martensite microstructure, as shown in Figure 9. The fine lath martensite can increase the surface area of the material, which is conducive to the formation of more corrosion protection layers, thus improving the corrosion resistance to a certain extent. At the same time, it can also reduce the stress concentration within the material, reducing the emergence of corrosion cracks and the expansion rate. Notably, the alloying elements away from the fusion zone were uniformly distributed and did not appear to be biased, which had a positive impact on the overall performance of the welded joint. Therefore, controlling the welding thermal cycle and temperature range is essential for preventing the precipitation of harmful phases and safeguarding the performance of welded joints.

### 3.4. Corrosion Resistance of the Welded Joints

**Figure 10:**
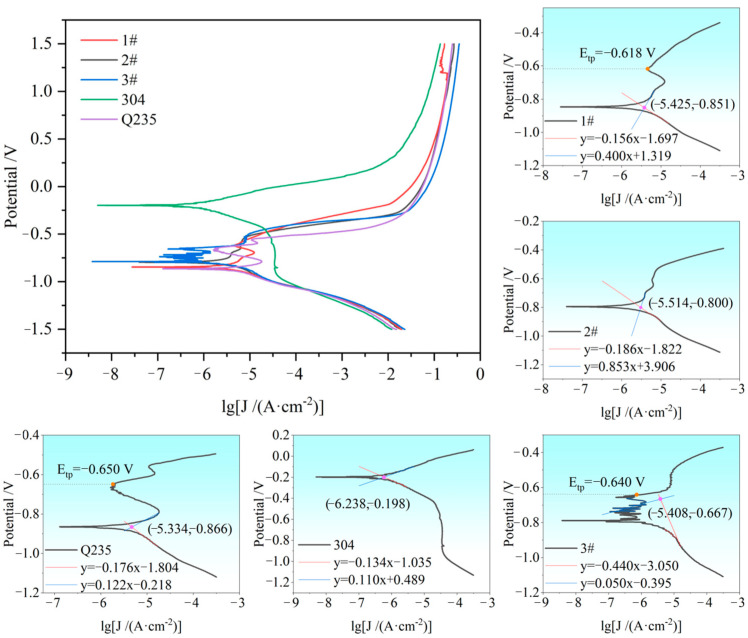
Potentiodynamic polarization curves of the samples.

Figure 10 shows the kinetic potential polarization curves of each sample in a 3.5 wt.% NaCl solution and their Tafel-zone-fitting data. The interval of ±60~120 mV from the open-circuit potential was regarded as the strong polarization zone, in which the corrosion current density and corrosion potential satisfy the Tafel relationship. The corresponding self-corrosion current (J_corr_) and self-corrosion potential (E_corr_) were obtained by using the Tafel extrapolation method for each sample, and the fitting data are shown in Table 2. The over-passivation potential was represented as E_tp_, i.e., the breakdown potential, which is the potential at which the corrosion current density increased rapidly with increasing anodic polarization potential. As shown in the dynamic potential polarization curves, the 304 stainless steel base material had no tendency to form a passivation film during electrochemical corrosion, whereas the other samples did. Unfortunately, the limited stable passivation zone of the curves suggested that other samples formed a passivation film during the process of electrochemical polarization but then quickly decomposed, and the film did not play a better role in corrosion resistance. From the fitted data in Table 2, it was evident that the self-corrosion current density of the 304 stainless steel was the smallest, at only 5.78 × 10^−7^ A·cm^−2^, whereas the Q235 base material presented the largest self-corrosion current density of 4.63 × 10^−6^ A·cm^−2^, which demonstrates that the 304 stainless steel had the best corrosion resistance, whereas the Q235 base material had the poorest corrosion resistance. Among the welded samples, the self-corrosion current density of 3# was 1.04 times greater than that of 1# and 1.28 times greater than that of 2#, which illustrated that the corrosion resistance of all the samples was 304 stainless steel > 2# > 1# > 3# > #Q235 base material, in descending order from the strongest to the weakest. Although the stable passivation zone of the samples during electrochemical polarization was minimal, the over-passivation potential could still be considered a reference for the corrosion resistance of the samples in this experiment. The breakdown potential of 2# was the highest, at −0.544 V, and that of the Q235 was the lowest, at −0.650 V, which indicates that the corrosion resistance of 1# was the best, whereas that of the Q235 base material was the worst. From the perspective of breakdown potential, the corrosion resistance was 2# > 1# > 3# > Q235 base material, in descending order from the strongest to the weakest (304 stainless steel did not form a significant passivation film, so there was no breakdown potential value). The Cr content in a grain is crucial to its corrosion resistance, and in principle, the higher the Cr content is, the greater the degree of corrosion resistance it possesses [21,22]. As indicated by Equations (1) and (2), the Cr equivalents in the weld region of samples 1#, 2#, and 3# were 3.7, 8.2, and 3.57, respectively. This suggested that 2# exhibited superior corrosion resistance compared with 1# and 3#, followed by 1#, which demonstrated an intermediate level of corrosion resistance, and 3# presented the poorest resistance. These observations aligned with the data of the kinetic potential polarization curves.

**Figure 11:**
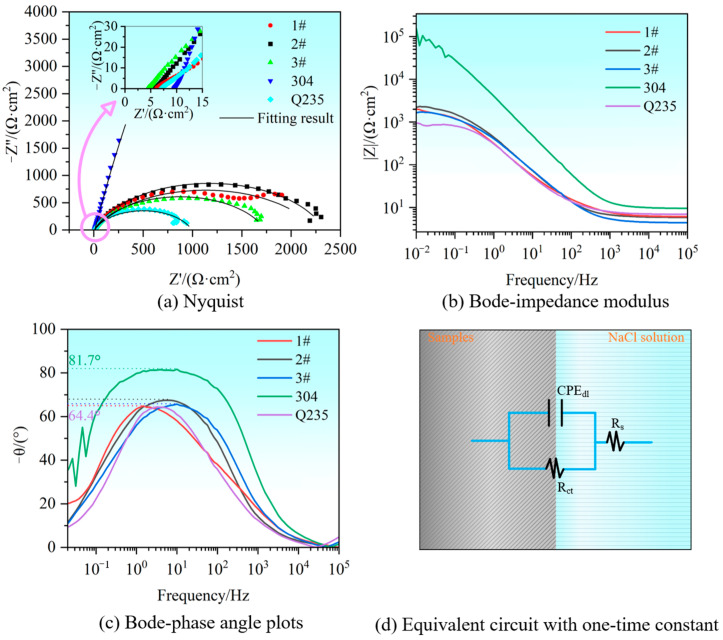
EIS test results of samples in 3.5 wt.% NaCl solution: (**a**) Nyquist plot; (**b**) Bode impedance modulus; (**c**) Bode-phase angle plots; (**d**) equivalent circuit.

To further investigate the corrosion resistance of the samples, an electrochemical impedance spectroscopy (EIS) test was conducted on each sample in a 3.5 wt.% NaCl solution. The data were fitted via ZSimpWin 3.60, and the results of the test and the corresponding equivalent circuit diagrams are presented in Figure 11. Figure 11a shows the Nyquist diagram, which allows the corrosion resistance of a material to be characterized by the magnitude of the capacitive loop radius, which is directly proportional to the aforementioned radius. It can be observed from the illustration that the 304 stainless steel sample corresponded to a considerably larger capacitive arc radius than the remaining samples did, but the Q235 base material exhibited the smallest one. Therefore, the corrosion resistance of the samples was 304 stainless steel > 2# > 1# > 3# > Q235 base material, in the order of strongest to weakest. Figure 11b shows a representation of the Bode impedance modulus plot. In general, materials with higher impedance moduli at low frequencies demonstrated superior corrosion resistance. The impedance modulus of 304 stainless steel was markedly different from that of the other samples, whereas that of the Q235 base material was the lowest but exhibited a slight proximity to that of the welded samples. This further substantiated the assertion that 304 stainless steel exhibited the most exemplary corrosion resistance, whereas the Q235 base material presented the least. Figure 11c shows the Bode phase angle plot. This demonstrated that with increasing frequency, the phase angle of the electrochemical polarization reaction exhibited a distinctive trend, initially increasing to a peak and then decreasing. This phenomenon was evident in all the samples, indicating that the passivation film produced on the sample surface during the polarization process was unstable, which is consistent with the results of the limited stable passivation zone exhibited by the kinetic potential polarization curves in Figure 10. The peaks of the curves in the middle- and low-frequency regions were distributed between 64.4° and 81.7°, all of which were less than 90°. This indicated that although the electrochemical reaction interface was still far from the state of ideal capacitance, the system still exhibited high impedance. (The value of the system impedance was determined by the combination of the Faraday impedance and the non-Faraday impedance.) The single peak of the curves in Figure 11c pointed to the existence of a single-time constant in the electrochemical reaction system. This result implied that the electrochemical reaction interface was characterized by the presence of only the charge-transfer process. Furthermore, the unstable passivation film, which corresponds to a brief interval of stable passivation on the kinetic potential polarization curves, exerted a negligible influence at the electrochemical reaction interface. Additionally, the electrochemical polarization process was not influenced by other processes, such as the adsorption of intermediates. Accordingly, the electrochemical impedance parameters could be fitted by the equivalent circuit illustrated in Figure 11d.

The principal electrochemical component parameters derived from the equivalent circuit (EEC) fitting are presented in Table 3. The fitting errors of all the electrochemical component parameter values listed in the table were less than 3%, and those of the majority of the parameters were controlled to within 1%. As shown in Figure 11a, the Nyquist diagram demonstrated that the equivalent circuit was accurately represented. Furthermore, the chi-square (χ^2^) analysis indicated a high degree of correlation between the fitted circuit and the experimental data. The aforementioned fitting parameters suggested that the selected equivalent circuit was reasonable and could be used to explain the electrochemical reaction process. R_s_ represents the solution resistance from the working electrode to the reference electrode, which corresponds to the starting position of the capacitive arc in Figure 11a. The resistance to charge transfer (R_ct_) is a measure of the difficulty of charge transfer across the electrode and electrolyte solution interface during the electrode reaction process. A smaller value of R_ct_ was indicative of more facile charge transfer and a higher corrosion rate. The data in Table 3 indicate that the charge-transfer resistance of 304 stainless steel was significantly greater than that of the other samples, reaching 95690 Ω·cm^2^. Conversely, the resistance of the Q235 base material was the lowest. The value of R_ct_ could be utilized to determine the order of corrosion resistance of the samples, with 304 stainless steel exhibiting the highest resistance, followed by the 2#, 1#, 3#, and Q235 base materials, in that order. The constant phase element (CPE) was employed to describe the double-layer capacitance (CPE_dl_), with n_dl_ representing the fitting parameter of the double-layer capacitance (0 < n < 1). When the electrodynamic potential was perturbed in the electrode system, a portion of the current was utilized to charge the double-layer capacitor (CPE_dl_), as previously described, and was designated the non-Faradaic current. The remaining portion of the current was employed directly for the electrode reaction, traversing the Faraday impedance (Z_F_) and adhering to Faraday’s law, and was classified as the Faraday current. The term R_p_ denotes the polarization resistance, which can be defined as the sum of all the impedances involved in the electrochemical polarization (activated polarization) process. Importantly, this value and the diffusion resistance of the substance (Z_W_) produced by the concentration polarization process together constitute the Faraday impedance (Z_F_). When calculating the polarization resistance, it was essential to consider all the impedances present in the activated polarization process. As evidenced by the preceding analysis, the electrochemical polarization system in this study encompassed solely the charge-transfer process. Consequently, the polarization resistance R_p_ was identical to the charge-transfer resistance R_ct_. In general, a higher value of polarization resistance indicates superior corrosion resistance. Therefore, the polarization resistance could be utilized to characterize the corrosion resistance of the sample, and the results were in accordance with those obtained from the kinetic potential polarization curve, i.e., the corrosion resistance of the sample was in descending order, beginning with 304 stainless steel, followed by 2#, 1#, and 3#, and conclusions were drawn with the Q235 base material.

## 4. Conclusions

This paper discusses in detail the metallographic microstructure and elemental distribution of the Q235 and 304 plate welds under different offset arcs, as well as the fusion characteristics and electrochemical corrosion performance of the 304 stainless steel side. The following conclusions are drawn:

The arc bias resulted in the enlargement of the heat-affected zone on the Q235 side, but the microstructure still consisted of ferrite and pearlite, and the pearlite content decreased with increasing proximity of the fusion line. Elemental scans revealed that Cr and Ni formed a significant concentration gradient near the fusion line, reflecting the mixing characteristics of the weld and the base metal. During mild steel welding, the fusion zone partially melts at high temperatures, forming coarse grains with an as-cast structure, which significantly affects the mechanical properties.

The side-heat-affected zone of 304 stainless steel is mainly composed of austenitic grains, and the fusion zone is narrow but prone to cracking. On the basis of the energy spectrometer test results and Schaeffler phase diagram analysis, the 1#, 2#, and 3# welds all formed lath-like martensite structures, which verified the accuracy of the Creq and Nieq calculations. The rapid cooling process of the weld promoted martensite phase transformation, whereas no alloying element segregation was observed in the weld; however, it should be noted that welded stainless steel joints may produce deleterious precipitates in the sensitization temperature range, which affects corrosion resistance.

Electrochemical tests revealed that the 304 stainless steel base material has the best corrosion resistance, whereas the Q235 material has the worst corrosion resistance. The welded joints with an arc bias toward the 304 side had the best corrosion resistance. In contrast, all the samples had limited stability of the passivation film formed during electrochemical polarization. The over-passivation potential can still be used as a reference for corrosion resistance, with sample 2# having the highest breakdown potential, indicating better corrosion resistance potential.

## Figures and Tables

**Table 1:** Chemical composition of the Q235 steel and SS 304 (wt%).

Element	C	Cr	Ni	Mn	S	P	Si	Fe
304	0.08	18–20.0	8–10.0	2	0.03	0.035	1	Bal
Q235	0.22	-	-	0.3	0.05	0.045	0.35	Bal

**Table 2:** E_corr_, J_corr_, and E_tp_ of samples with different welding conditions.

Samples	1#	2#	3#	304	Q235
E_corr_ (V)	−0.851	−0.800	−0.667	−0.198	−0.866
J_corr_ (A/cm^2^)	3.76 × 10^−6^	3.06 × 10^−6^	3.91 × 10^−6^	5.78 × 10^−7^	4.63 × 10^−6^
E_tp_ (V)	−0.618	−0.544	−0.640	/	−0.650

**Table 3:** Fitting electrochemical parameters of the samples.

Samples	R_s_/ (Ω·cm^2^)	CPE_dl_/ (S·sec^ndl^·cm^−2^)	n_dl_	R_ct_/ (Ω·cm^2^)	R_p_/ (Ω·cm^2^)	χ^2^
1#	6.393	8.08 × 10^−4^	0.732	2249	2249	7.34 × 10^−3^
2#	5.928	5.01 × 10^−4^	0.804	2335	2335	6.59 × 10^−4^
3#	4.404	5.50 × 10^−4^	0.779	1737	1737	1.20 × 10^−3^
304	9.803	4.74 × 10^−5^	0.911	95690	95690	2.82 × 10^−3^
Q235	7.265	7.10 × 10^−4^	0.789	985.5	985.5	5.02 × 10^−3^

## Data Availability

The original contributions presented in the study are included in the article, further inquiries can be directed to the corresponding author.
